# Comparative Evaluation of HPV PLUS ELITe MGB® and Allplex™ HPV HR Assays for High‐Risk Human Papillomavirus Genotyping in Cervical Samples

**DOI:** 10.1002/jmv.70954

**Published:** 2026-05-16

**Authors:** Sarah Mafi, Julie Delpont, Amandine Bigot, Sébastien Hantz

**Affiliations:** ^1^ Department of Bacteriology, Virology and Hygiene Limoges University Hospital Limoges France; ^2^ University of Limoges, INSERM, RESINFIT Limoges France

**Keywords:** Allplex™ HPV28, cervical samples, comparative study, ELITe MGB®, high‐risk HPV, HPV genotyping

## Abstract

To compare the performance of the HPV PLUS ELITe MGB® (ELITechGroup) and Allplex™ HPV HR (Seegene) assays for the detection and genotyping of high‐risk HPV (HR‐HPV) in cervical samples. HPV typing results using both techniques were obtained for 491/500 cervical samples. The ELITe assay provides individual detection of HPV16, 18, 31 and 45, and pooled detection of other HR‐HPV into HR1 (33/52/58) and HR2 (35/39/51/56/59/66/68), whereas the Allplex™ assay provides individual detection of all HR‐HPV types. Cytological data were available for 283 samples. Concordance, percent agreements, and kappa coefficient were calculated. Ct values, discordances, and associations with cytology were analyzed. Overall agreement was 88.6% (*κ* = 0.77, substantial). HPV DNA was detected in 40.5% (199/491) of samples with the ELITe assay and 47.9% (235/491) with the Allplex™ assay. The Allplex™ assay showed significantly higher detection than the ELITe assay for HPV16 (50 vs. 36), HPV18 (37 vs. 26), and HR1 genotypes (66 vs. 48, particularly HPV52). Most ELITe − /Allplex+ cases occurred in NILM and ASCUS samples (58% showing Ct ≤ 35), but with no significant difference regardless of cytology. The Allplex™ assay demonstrated higher analytical sensitivity for clinically relevant genotypes. This increased detection could reflect earlier identification before lesion onset or, conversely, detection of transient infections of uncertain clinical relevance, underscoring the need to balance analytical performance with clinical utility.

## 1 Introduction

Cervical cancer represents a major global health challenge, ranking as the fourth most frequently diagnosed cancer and the fourth leading cause of cancer‐related death in women worldwide [1]. Persistent genital infection with a carcinogenic human papillomavirus (HPV) genotype is a necessary but not sufficient cause of invasive cervical cancer [2]. The human papillomavirus family comprises over 200 genotypes, of which approximately 40 are known to infect the mucosal epithelium [2]. Genital HPV types are classified as low‐risk (LR‐HPV), primarily associated with benign lesions such as genital warts, or high‐risk (HR‐HPV), which are responsible for cervical intraepithelial neoplasia, including high‐grade lesions (CIN2/3), and invasive carcinoma [3]. According to the International Agency for Research on Cancer (IARC), 12 HPV types (16/18/31/33/35/39/45/51/52/56/58/59) are currently classified as carcinogenic to humans (Group 1), and one additional genotype (HPV68) is considered probably carcinogenic (Group 2 A) [4]. HPV16 and 18 account for the majority of cervical cancer cases, being responsible for at least 70% of cervical cancers [5, 6]. Furthermore, multiple HR‐HPV type infections are frequently observed in cervical specimens. Although their clinical significance remains partially elucidated, several studies suggest that multiple infections may contribute to viral persistence and influence the risk of cervical neoplasia progression [7, 8, 9].

A wide range of molecular approaches and platforms for HPV detection and genotyping have been developed and are now routinely implemented in cervical cancer screening, with HR‐HPV testing increasingly used as a primary screening tool in many countries [10, 11, 12, 13]. Compared with cytology, HR‐HPV testing has demonstrated higher sensitivity for the detection of high‐grade cervical lesions (CIN2 +), allowing earlier identification of women at risk of cervical cancer [14, 15]. In many screening strategies, HR‐HPV‐positive women are subsequently triaged using cytology and/or partial genotyping, particularly for HPV16 and 18, which are associated with the highest oncogenic risk.

In recent years, the number of commercially available HPV molecular assays has increased substantially, with more than 250 distinct tests and over 500 variants reported worldwide as of December 2023 [16]. These assays exhibit varying levels of analytical sensitivity and genotyping resolution, which may influence their clinical performance and interpretation.

Only clinically validated HPV tests should be used in cervical cancer screening. International validation criteria have been established to define the requirements for HPV DNA assays, including clinical sensitivity, specificity, and reproducibility, as well as inter‐laboratory agreement. These criteria, initially proposed by Meijer et al. [17] and further developed through international initiatives such as VALGENT [18], are now commonly used as a benchmark for assay validation. More recent guidelines have introduced criteria for second‐generation comparator tests, including more detailed resolution of HPV genotypes and requirements for consistent non‐inferior sensitivity for CIN2+ and CIN3+ and specificity for ≤ CIN1 compared with first‐generation comparator tests [19].

The HPV PLUS ELITe MGB® assay (ELITechGroup, Bruker Corporation, United States) has been recently introduced to the market and provides partial genotyping with individual detection of HPV16, 18, 31, and 45, and grouped detection of other HR‐HPV types. In parallel, the Allplex™ HPV HR assay (Seegene, Korea) allows full genotyping of all targeted HR‐HPV types and has been recently evaluated against established validation criteria, demonstrating compliance with international requirements for clinical sensitivity, specificity, and reproducibility for primary cervical cancer screening [20]. Although both assays are used in clinical and laboratory settings, differences in analytical sensitivity and genotyping strategies may impact their clinical relevance. Comparative evaluation of these assays is therefore essential to assess their performance and potential implications for clinical management.

In this context, this study aims to compare the performance of the HPV PLUS ELITe MGB® assay (ELITe) and the Allplex™ HPV HR assay (Allplex^TM^ HR) for the detection and genotyping of HR‐HPV, using a panel of cervical samples.

## 2 Materials and Methods

### 2.1 Sample Collection

**Figure 1:**
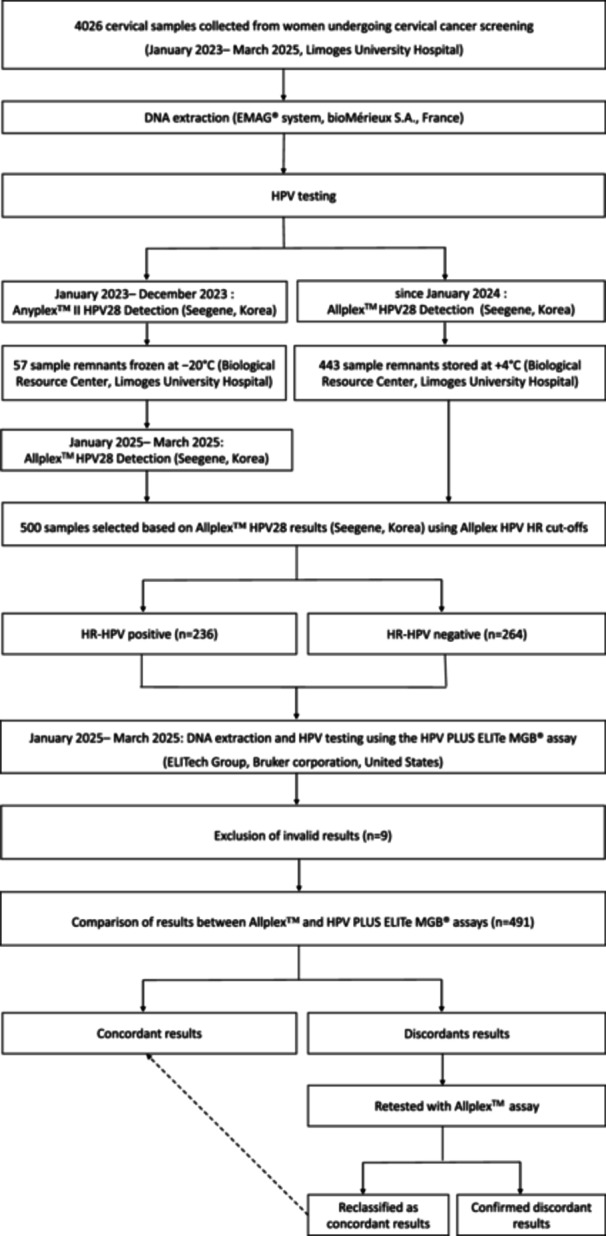
Flow chart of the study design.

The study was conducted at the Virology Department, Limoges University Hospital, France. Cervical samples collected in ThinPrep® Pap Test PreservCyt solution (Hologic, USA) from women undergoing cervical cancer screening between January 2023 and March 2025 were retrospectively selected to construct a panel of 500 samples for analysis (Figure 1). Among these 500 samples, 57 were collected in 2023 and 443 in 2024–2025.

Samples collected in 2023 had been initially tested using the Anyplex™ II HPV28 assay as part of routine laboratory practice. Sample remnants from 2023 were stored at −20°C in the CRBioLim biobank (ISO 20387 certified). From 2024 onward, the Anyplex™ II HPV28 assay was replaced by the Allplex™ HPV28 assay in our laboratory workflow. Samples collected in 2024–2025 were therefore routinely tested with the Allplex™ HPV28 assay and stored at +4°C until subsequent analysis. Samples initially tested using Anyplex™ II HPV28 assay were retested using the Allplex™ HPV28 assay between January and March 2025 to ensure a consistent comparison of techniques. All selected samples (*n* = 500) were subsequently analyzed between January and March 2025 using the HPV PLUS ELITe MGB® assay.

Among the 500 selected samples, 236 tested positive for at least one HR‐HPV type based on Allplex™ HPV28 results: 205 were single infections and 31 multiple infections. To ensure representation of each genotype, the panel included ≥ 27 samples for each individually detected HR‐HPV type (HPV16/18/31/45) and ≥ 7 for pooled groups (HR1 and HR2). The corresponding genotype distribution used for sample selection is detailed in Supplementary Table 1. The remaining 264 samples were HR‐HPV‐negative.

Primer set A of the Allplex™ HPV28 assay is identical to that of the clinically validated Allplex™ HPV HR assay. To ensure a clinically relevant comparison, results were interpreted using the genotype‐specific Ct (cycle thresholds) established for the Allplex™ HPV HR assay: rather than the default Ct ≤ 43 (Allplex™ HPV 28 assay), results are considered positive if Ct ≤ 40 for HPV16 and HPV18, Ct ≤ 37 for HPV31/33/45/52/58, and Ct ≤ 35 for HPV35/39/51/56/59/66/68. Therefore, the analysis performed using the Allplex™ HPV28 assay is equivalent to a comparison between the Allplex™ HPV HR and ELITe assays. Samples were considered negative if no HPV was amplified or if amplification occurred above the genotype‐specific Ct threshold, provided that the internal control (IC) was amplified (Ct ≤ 43).

To assess a potential storage‐related effect, all ELITe/Allplex discordant results were systematically retested using the Allplex™ assay with sample remnants stored under the same conditions as for ELITe testing. If the retesting result was consistent with the initial Allplex™ result, the discordance was considered confirmed. If the retesting result differed from the initial Allplex™ result, the retest result was retained for analysis. Retesting was performed using the same assay, extraction method, and interpretation criteria as in the initial analysis.

### 2.2 HPV Testing

#### 2.2.1 Allplex™ Assay

DNA was extracted from 800 µL of each sample using the EMAG® system (bioMérieux S.A., France), with a final elution volume of 80 µL. This extraction platform, routinely used at Limoges University Hospital for various molecular analyses, was internally validated before use, as it is not listed in the instructions for use.

The Allplex™ HPV28 assay is a multiplex real‐time PCR kit designed to simultaneously amplify, detect, and differentiate 28 HPV types in a single reaction. It incorporates Seegene's proprietary technologies, including DPO™ (Dual Priming Oligonucleotide), TOCE™ (Tagging Oligonucleotide Cleavage and Extension), and MuDT™ (Multiple Detection Temperature), enabling precise genotyping and semi‐quantitative analysis.

Primer set A targets 14 HPV types: 12 classified as Group 1 carcinogenic (HPV16/18/31/33/35/39/45/51/52/56/58/59), one probably carcinogenic (HPV68, Group 2 A), and one possibly carcinogenic (HPV66, Group 2B). Primer set B detects 14 additional HPV types (6/11/26/40/42/43/44/53/54/61/69/70/73/82) classified as Group 2B or Group 3.

Briefly, 5 µL of extracted DNA were added to 15 µL of the reaction mixture for amplification with primer set A. PCR set‐up was performed using the ESTREAM® pipetting instrument (bioMérieux S.A., France), and DNA amplification was carried out on the CFX96™ real‐time PCR system (Bio‐Rad, USA), according to the manufacturer's instructions. The human β‐globin gene (IC) was co‐amplified with the HPV L1 gene targets. Data were automatically analyzed using Seegene software.

#### 2.2.2 HPV PLUS ELITe MGB® Assay

The ELITe assay is a qualitative multiplex real‐time PCR test detecting 14 HPV types, with individual identification of HPV16, 18, 31, and 45, and grouped detection of HR1 (HPV33/52/58) and HR2 (HPV35/39/51/56/59/66/68). DNA extraction and amplification were performed using the ELITe BeGenius® system (ELITechGroup, Bruker Corporation, USA), an automated platform integrating nucleic acid extraction and real‐time PCR.

For each sample, 200 µL was processed according to the manufacturer's instructions, with a final elution volume of 100 µL. Twenty microliters of extracted DNA were added to 20 μL of the PCR mix. Amplification was carried out using the HPV PLUS PCR Mix, which contains primers and probes incorporating ELITe MGB® and TaqMan® MGB technologies. Fluorescence signals were automatically analyzed by the BeGenius® software and expressed as Ct values.

A result was considered positive when the Ct for a given target was < 45. Samples were considered negative if no HPV amplification was observed or if amplification occurred at a Ct ≥ 45, provided that the IC was amplified (Ct < 35). Specificity of positive signals was confirmed by melting curve analysis.

### 2.3 Liquid‐Based Cytology

Cervical liquid‐based cytology (LBC) was routinely performed after HPV genotyping, following the French Haute Autorité de Santé (HAS) screening algorithm [21]. Slides were stained according to the standard laboratory protocol, and cytological diagnoses were performed by experienced pathologists. Results were classified according to the 2014 Bethesda System [22]. Cytological results were retrospectively extracted from medical records for the comparative evaluation of the Allplex™ and ELITe assays.

### 2.4 Statistical Analysis

HPV prevalence was determined according to the ELITe assay classification, which reports four individual genotypes (HPV16/18/31/45) and two grouped categories (HR1, HR2). Analyses were performed both overall and by genotype. Agreement between the Allplex™ and ELITe assays was assessed using overall, positive, and negative percent agreement, and kappa coefficient (*κ*). *κ* values were interpreted as follows: ≤ 0.00, no agreement; 0.01–0.20, slight; 0.21–0.40, fair; 0.41–0.60, moderate; 0.61–0.80, substantial; 0.81–0.99, almost perfect; and 1.00, perfect agreement. The McNemar test was used to determine whether differences in HPV detection between assays were statistically significant for paired data. Discordant results (ELITe − /Allplex + ; ELITe + /Allplex − ) were analyzed by genotype, cytology, and Ct values. Ct comparisons between assays included only concordant positive samples, excluding HR1/HR2 multiple infections, and were evaluated by linear regression and Bland–Altman analysis. Normality was assessed using the Shapiro–Wilk test, and Ct values of the IC were compared between concordant and discordant samples using the Mann–Whitney *U* test when non‐normal distribution was observed. All analyses were conducted with Prism 9 (GraphPad, USA) and BiostaTGV; *p* < 0.05 was considered significant.

## 3 Results

### 3.1 Overall HPV Prevalence

Among the 500 cervical samples initially collected, 9 (1.8%) were invalid by the ELITe assay, resulting in 491 samples available for analysis (Figure 1). No invalid results were observed with the Allplex™ assay, as only valid Allplex™ results were selected for comparison, and no invalid results occurred during retesting of discordant samples. The 9 invalid ELITe results were all obtained from samples collected between January 2024 and March 2025, which had been stored at +4°C before ELITe testing.

For the 14 HPV types targeted by both assays, HPV DNA was detected in 40.5% (199/491) of samples with the ELITe assay and 47.9% (235/491) with the Allplex™ assay (Table 1). Overall agreement was 88.6% (435/491), with positive and negative percent agreements of 80.4% (189/235) and 96.1% (246/256), respectively. The kappa coefficient was 0.77, indicating substantial agreement. HPV detection differed significantly between assays (*p* < 0.0001, McNemar's test for paired data). In addition, for HPV16, the overall agreement reached 97.15% (*κ* = 0.82), indicating almost perfect concordance, although the detection rate was significantly higher with the Allplex™ assay (Table 2).

**Table 1:** Comparison of the ELITe MGB® and Allplex^TM^ assays for HPV DNA detection.

		ELITe MGB®		
		Positive	Negative	Total
**Allplex™**	**Positive**	189 (38.5%)	46 (9.4%)	235 (47.9%)
	**Negative**	10 (2.0%)	246 (50.1%)	256 (52.1%)
	**Total**	199 (40.5%)	292 (59.5%)	491 (100%)

**Table 2:** Genotype‐specific comparison of HPV detection (HPV16, 18, 31, 45, HR1, and HR2) between the ELITe MGB® and Allplex™ assays.

			Genotype‐sample combinations (*N* = 491*)									
	Eli +	All +	Eli + /All +	Eli + /All‐	Eli‐/All +	Eli‐/All‐	Overall agreement (%)	PPA (%)	NPA (%)	*κ*	*Int.*	*p*
	*n* (%)	*n* (%)	*n*	*n*	*n*	*n*						
HPV16	36 (7.33)	50 (10.18)	36	0	14	441	97.15	72.00	100	0.82	Almost perfect	< 0.001
HPV18	26 (5.30)	37 (7.54)	26	0	11	454	97.76	70.27	100	0.81	Almost perfect	< 0.01
HPV31	38 (7.74)	35 (7.13)	32	6	3	450	98.17	91.43	98.68	0.87	Almost perfect	0.50
HPV45	24 (4.89)	27 (5.50)	23	1	4	463	98.98	85.19	99.78	0.90	Almost perfect	0.37
HR1	48 (9.78)	66 (13.44)	47	1	19	424	95.93	71.21	99.76	0.80	Substantial	< 0.001
HR2	55 (11.20)	50 (10.18)	44	11	6	430	96.54	88.00	97.51	0.82	Almost perfect	0.33

*Note:* Eli +/All +, positive with both assays; Eli +/All −, ELITe MGB® positive and Allplex^TM^ negative; Eli −/All +, ELITe MGB® negative and Allplex^TM^ positive; Eli −/All −, negative with both assays; NPA, negative percent agreement; PPA, positive percent agreement; *K*, kappa coefficients; Int., Interpretation of kappa coefficients (*K*); *p*, McNemar's test. Bold *p*‐values (< 0.05) indicate a statistically significant difference in detection between assays.

* A total of 9 samples with invalid results using the ELITe MGB® assay were excluded from the analysis, resulting in 491 cervical samples analyzed.

### 3.2 Comparison of Ct Values Between Assays

Among the 189 samples positive with both assays, 208 concordant HR‐HPV types were identified: 36 HPV16, 26 HPV18, 32 HPV31, 23 HPV45, 47 HR1 types, and 44 HR2 types (Table 2). In the HR1 group, 46 were single infections and one was multiple. In the HR2 group, 36 were single infections and 8 were multiple. As the ELITe assay detects HR1 and HR2 as pooled targets, Ct comparisons were restricted to single infections in these groups, while all concordant detections (single or multiple) were considered for HPV16, 18, 31, and 45. Overall, 199 genotype‐level pairs from 189 samples were analyzed.

**Figure 2:**
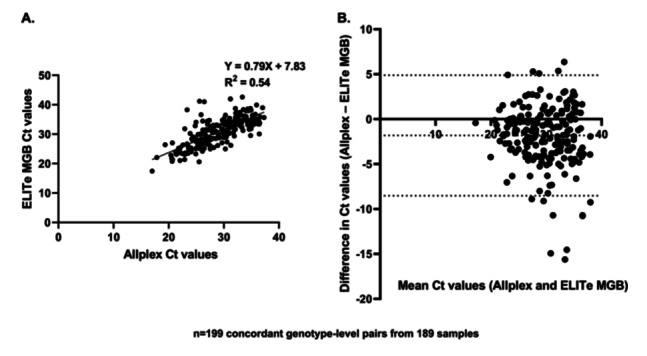
Comparison of Ct values between the ELITe MGB® and Allplex™ assays, when both assays were positive. A total of 189 samples, corresponding to 199 concordant genotype‐level pairs, were included in the analysis. Ct comparisons included all concordant detections of HPV16, HPV18, HPV31, and HPV45 (individually detected by the ELITe assay), while only single infections were considered for HR1 and HR2 groups, which are detected as pooled targets by the ELITe assay. (A) Linear regression analysis of Ct values obtained with both assays. The regression line is shown with the corresponding equation. (B) Bland–Altman plot representing the differences in Ct values between the Allplex™ and ELITe MGB® assays (Allplex – ELITe MGB) against the mean Ct values. Dashed lines represent the mean difference and the 95% limits of agreement (mean ± 1.96 SD).

Linear regression (Figure 2A) on these 199 pairs showed a significant correlation between assays (*R*² = 0.54, *p* < 0.0001), indicating moderate agreement. Bland–Altman analysis (Figure 2B) revealed a mean Ct difference of −1.93  ±  3.43, indicating that Ct were generally lower with the Allplex™ assay. The 95% limits of agreement ranged from −8.65 to +4.79, with 14/199 pairs (7.0%) outside this range.

### 3.3 Comparison of HPV Detection for Individual Genotypes (HPV16/18/31/45) and Grouped Genotypes (HR1 and HR2)

Overall agreement between assays ranged from 95.93% to 98.98% for HPV16, 18, 31, 45, HR1, and HR2 (Table 2). Positive and negative percent agreements were ≥ 70.27% and ≥ 97.51%, respectively. Agreement was almost perfect for HPV16, 18, 31, 45, and HR2 (*κ* ≥ 0.81), and substantial for HR1 (*κ* = 0.80).

Seventy‐six discordant results were observed in 73/491 samples (14.9%). Most discordances (75.0%, 57/76) corresponded to samples positive with the Allplex™ assay but negative with the ELITe assay (ELITe − /Allplex + ), and 25.0% (19/76) corresponded to samples negative with Allplex™ but positive with ELITe (ELITe + /Allplex‐).

**Figure 3:**
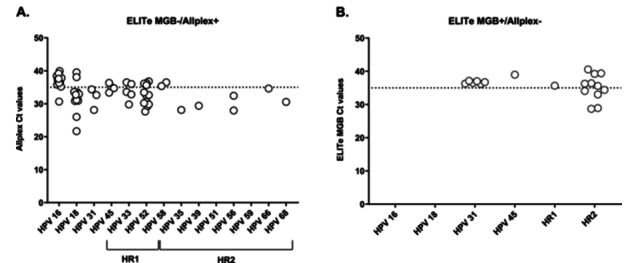
Ct value distribution for discordant results between the ELITe MGB® and Allplex™ assays, stratified by HPV type. A dashed line at Ct = 35 was added to approximate the distinction between higher and lower viral loads. (A) Ct values for genotypes detected by the Allplex™ assay but not by the ELITe MGB® assay (ELITe MGB − /Allplex + ). (B) Ct values for genotypes detected by the ELITe MGB® assay but not by the Allplex™ assay (ELITe MGB + /Allplex‐).

Among ELITe − /Allplex+ cases, genotypes concerned were: HPV16 (*n* = 14), HPV52 (*n* = 12), HPV18 (*n* = 11), HPV33 (*n* = 5), HPV45 (*n* = 4), HPV31 (*n* = 3), HPV58 (*n* = 2), HPV56 (*n* = 2), and isolated cases of HPV35, HPV39, HPV66, and HPV68 (n = 1 each) (Table 2, Figure 3A). Detection rates for HPV16, HPV18, and HR1 were significantly higher with Allplex™ than with ELITe (*p* < 0.05). No significant differences were observed for HPV31, HPV45, or HR2, although HPV31 and HR2 were slightly more often detected with ELITe.

Relative to Allplex™ detections for each target, ELITe − /Allplex+ discordances were most frequent for HPV52 (12/26, 46.2%), followed by HPV18 (11/37, 29.7%), HPV56 (2/7, 28.6%), HPV16 (14/50, 28.0%) and HPV33 (5/21, 23.8%) (Table 2, Supplementary Table 1).

Among ELITe + /Allplex− discordances, the genotypes involved were HR2 (11/19), HPV31 (6/19), HPV45 (1/19), and HR1 (1/19) (Table 2, Figure 3B). Of these, 10 samples showed HPV amplification with the Allplex™ assay but with Ct values above the positivity thresholds, and were therefore interpreted as negative. These included HPV31 (5/10), HPV45 (1/10), HR1 (HPV33; 1/10), and HR2 (HPV35, HPV51, and HPV68; 3/10).

### 3.4 Cytological Analysis of Cervical Samples

**Figure 4:**
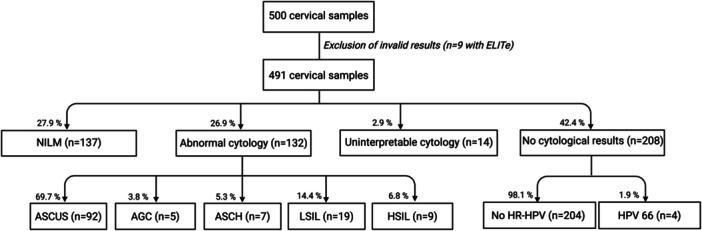
Distribution of cervical cytology results in the study population. NILM (negative for intraepithelial lesion or malignancy), ASCUS (atypical squamous cells of undetermined significance), AGC (atypical glandular cells), ASCH (atypical squamous cells, cannot exclude high‐grade squamous intraepithelial neoplasia), LSIL (low‐grade squamous intraepithelial lesion), HSIL (high‐grade squamous intraepithelial lesion).

NILM (negative for intraepithelial lesion or malignancy) was reported in 27.9% (137/491) of the total samples, while abnormal cytological findings were observed in 26.9% (132/491) (Figure 4). Among the samples with abnormal cytology (*n* = 132), 69.7% (92/132) were classified as ASCUS (atypical squamous cells of undetermined significance), 3.8% (5/132) as AGC (atypical glandular cells), 5.3% (7/132) as ASCH (atypical squamous cells, cannot exclude high‐grade squamous intraepithelial neoplasia), 14.4% (19/132) as LSIL (low‐grade squamous intraepithelial lesion), and 6.8% (9/132) as HSIL (high‐grade squamous intraepithelial lesion). No invasive cervical cancer was identified. Cytology results were uninterpretable in 2.9% (14/491) of cases. The remaining 42.4% (208/491) had no cytological data, either because no HPV had been detected by the Allplex™ assay or, in cases of HPV66, which were not referred for cytology, as it is currently classified as possibly carcinogenic to humans (Group 2B) with no attributable fraction for cervical cancer [4].

### 3.5 Analysis of Discordant Results by Cytological Grade

Fifty‐seven ELITe − /Allplex+ discordances were found in 55 cervical samples. Among NILM cases, 27 discordances occurred in 26/137 samples (19.0%), and among ASCUS cases, 20 discordances occurred in 19/92 samples (20.7%). Other discordances were found in 1/5 AGC (20.0%), 1/7 ASCH (14.3%), and 2/19 LSIL (10.5%) samples. None were observed in HSIL. Five discordances involved samples with uninterpretable cytology (5/14, 35.7%), and one was associated with a sample lacking cytological data (HPV66 detection).

Nineteen ELITe + /Allplex− discordances were observed in 19 samples, including 8/137 NILM (5.8%), 5/92 ASCUS (5.4%), 1/19 LSIL (5.3%), and 1/9 HSIL (11.1%) involving an HPV from the HR2 group. None were found in AGC (0/5) or ASCH (0/7). Two occurred in uninterpretable cytology (2/14, 14.3%), and two in samples without cytological results (HR‐HPV negative by Allplex™).

### 3.6 Analysis of HPV DNA Levels (Ct Values) in Discordant Results by Genotype and Cytological Grade

**Figure 5:**
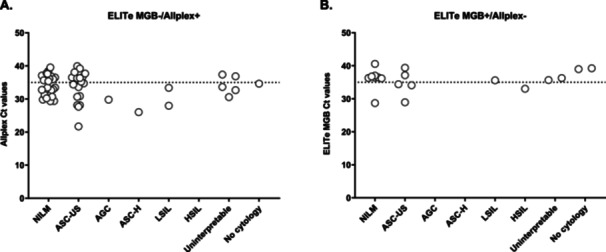
Ct value distribution for discordant results according to cytological grade. NILM (negative for intraepithelial lesion or malignancy), ASCUS (atypical squamous cells of undetermined significance), AGC (atypical glandular cells), ASCH (atypical squamous cells, cannot exclude high‐grade squamous intraepithelial neoplasia), LSIL (low‐grade squamous intraepithelial lesion), HSIL (high‐grade squamous intraepithelial lesion). A dashed line at Ct = 35 was added to approximate the distinction between higher and lower viral loads. (A) Ct values for samples positive with the Allplex™ assay but negative with the ELITe MGB® assay (ELITe MGB − /Allplex + ), distributed by cytological classification. (B) Ct values for samples positive with the ELITe MGB® assay but negative with the Allplex™ assay (ELITe MGB + /Allplex‐), distributed by cytological classification. Samples without cytology were not referred for cytological evaluation, as they tested negative with the Allplex™ assay or, in the case of HPV66, as it is currently classified as possibly carcinogenic to humans (Group 2B) with no attributable fraction for cervical cancer [4].

Among the 57 ELITe − /Allplex+ discordances, 33 (57.9%) had Ct ≤ 35, suggesting that the ELITe assay failed to detect some samples with moderate to high HPV DNA levels (Figure 3A). When stratified by genotype, all HPV types showed a majority of discordant cases with Ct ≤ 35, except for HPV16 and HPV58, for which most cases had Ct > 35 (13/14 and 2/2 cases, respectively). Based on cytological classification, NILM (*n* = 27) and ASCUS (*n* = 20) samples showed a comparable distribution of Ct ≤ 35 and > 35 (15 vs. 12 for NILM, and 10 vs. 10 for ASCUS, respectively) (Figure 5A). Discordances involving higher‐grade abnormalities—AGC (*n* = 1), ASCH (*n* = 1), and LSIL (*n* = 2)—were all associated with Ct ≤ 35 with the Allplex™ assay, indicating relatively high viral DNA levels in these samples. Among samples with uninterpretable cytology (*n* = 5), three had Ct ≤ 35 and two had Ct > 35. The discordant sample lacking cytological data (HPV66 detection) showed a Ct close to 35 (Ct = 34.62).

Among the 19 ELITe + /Allplex‐ discordances, most samples (14/19, 73.7%) had Ct > 35 with the ELITe assay (Figure 3B), indicating that these discrepancies mainly involved samples with low HPV DNA levels. For 10 of these 19 samples, HPV amplification was observed with the Allplex™ assay, but with Ct values exceeding the positivity thresholds, while the remaining cases showed no detectable amplification. All genotypes showed only discordant cases with Ct > 35, except for HR2, for which 54.6% of cases (6/11) had Ct > 35. Regarding cytological classification, most NILM samples (7/8, 87.5%) had Ct > 35 (Figure 5B). Among ASCUS samples (*n* = 5), the distribution was balanced, with three cases ≤ 35 and two > 35. The LSIL sample had a Ct > 35, while the HSIL sample showed a Ct < 35. All discordant cases with uninterpretable cytology (*n* = 2) and those without cytological data (*n* = 2) had Ct > 35.

### 3.7 Analysis of Discordant Results According to Sample DNA Quality

**Figure 6:**
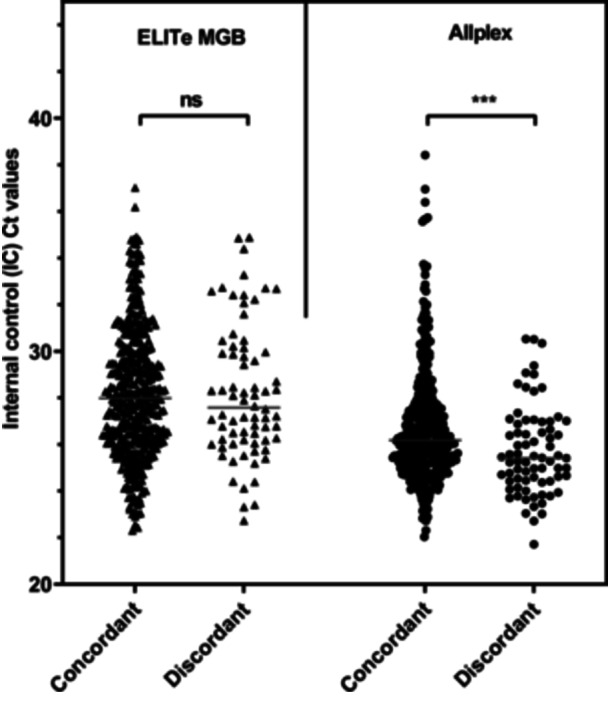
Comparison of internal control (IC) Ct values between concordant and discordant samples. (A) ELITe MGB® assay. (B) Allplex™ assay. Each point represents the Ct value of the internal control (IC) for a given sample. Bars indicate median values. Statistical comparisons were performed using the Mann–Whitney *U* test. No significant difference was observed for the ELITe MGB® assay (ns), while a significant difference was found for the Allplex™ assay between concordant and discordant samples (***p < 0.001).

To assess whether sample DNA quality influenced assay performance, Ct values of the IC were compared between concordant and discordant results for each assay (Figure 6A,B). Distributions failed the Shapiro–Wilk test (*p* < 0.05), so medians were compared using the Mann–Whitney *U* test.

For the ELITe assay, median IC Ct did not differ significantly between concordant (27.98 cycles [IQR 26.15–29.94]) and discordant (27.58 cycles [IQR 26.18–30.10]) samples (*p* = 0.98), suggesting similar DNA quality. In contrast, for the Allplex™ assay, concordant samples showed a statistically higher median IC Ct than discordant samples (26.18 cycles [IQR 25.21–27.53] vs. 25.41 cycles [IQR 24.42–26.92]; *p* = 0.0003).

## 4 Discussion

Accurate detection and genotyping of HR‐HPV are essential for cervical cancer prevention and management. Given the range of commercial assays with differing performance characteristics, comparative evaluations are crucial to identify optimal diagnostic tools suited for clinical use and surveillance. This study compared the HPV PLUS ELITe MGB® assay with the Allplex™ HPV HR assay. To our knowledge, this is the first study evaluating the performance of the HPV PLUS ELITe MGB® assay. Allplex™ HPV28 results were interpreted using the clinically validated thresholds defined for the Allplex™ HPV HR assay, which shares the same primer set A as the Allplex™ HPV28 assay.

Substantial agreement was observed between the assays (*κ* = 0.77), and a significantly higher overall detection rate was reported with the Allplex™ assay. HPV16, HPV18, HPV31, and HPV45—genotypes individually reported by the ELITe assay—exhibited almost perfect agreement between assays (*κ* ≥ 0.81). The HR2 group also demonstrated almost perfect agreement, whereas agreement for the HR1 group remained substantial. Detection rates for HPV16, HPV18, and the HR1 group were significantly higher with the Allplex™ assay, suggesting increased analytical sensitivity for these targets. Among 76 discordant results (15% of samples), 75% were ELITe − /Allplex+ cases, most frequently involving HPV52, HPV18, HPV56, and HPV16. HPV52, within HR1, may partly explain lower concordance in this group. Regarding the ELITe + /Allplex− discordances (approximately 25% of all discordant genotype‐sample combinations), HPV31 and the HR2 group were more frequently detected by the ELITe assay (6 vs. 3 and 11 vs. 6 detections, respectively). Although not statistically significant, this trend may indicate improved detection of these genotypes by the ELITe assay. In a recent study, Mafi *et al*. compared the Allplex™ HPV28 and Anyplex™ II HPV28 assays and reported that the Allplex™ assay primers may provide enhanced detection sensitivity for HPV51 and HPV52, whereas the Anyplex™ II assay showed better detection for HPV31 and HPV39 [23]. These findings corroborate the observed improved detection of HPV52 by the Allplex™ assay and support the hypothesis of a reduced detection performance for HPV31 with the Allplex™ assay, reflecting differential analytical performance between assays. The observed differences may be explained by intrinsic variations in assay design—such as primer and probe design or target region specificity for each genotype—although these technical parameters remain proprietary.

Most ELITe − /Allplex+ discordant results occurred in samples with NILM and ASCUS cytology (approximately 19% and 21% of cases, respectively), followed by AGC (20%), ASCH (14%), and LSIL (11%). No discordant case was found in HSIL samples. This distribution suggests that differences in assay sensitivity may be more likely to emerge in samples with lower viral loads, particularly those with NILM and ASCUS cytology. However, the small number of samples in the ASCH (*n* = 7), AGC (*n* = 5), and HSIL (*n* = 9) categories limits interpretation. In addition, Ct analysis does not fully support the hypothesis that discordances are primarily linked to low viral loads: 58% of ELITe − /Allplex+ discordances exhibited Ct ≤ 35. Notably, all ELITe − /Allplex+ discordances observed in AGC, ASCH, and LSIL categories also exhibited Ct values within this range. Conversely, most ELITe + /Allplex‐ discordances had Ct > 35 (74%), indicating that these discrepancies mainly involved samples with low HPV DNA levels. In about half of these cases, HPV was detected by the Allplex™ assay but with Ct values above the clinical positivity thresholds, while in the others, no amplification was observed. This suggests that although the Allplex™ assay shows higher analytical sensitivity overall, the use of predefined interpretation thresholds may lead to the classification of some low‐level infections as negative. These results indicate that factors beyond viral load, such as assay‐specific characteristics, likely contribute to detection discrepancies.

Clinically, higher analytical sensitivity, as observed overall with the Allplex™ assay, may facilitate earlier identification of HPV infections [24, 25] in women aged 30–65 years with NILM cytology, potentially warranting retesting after 1 year rather than five, as recommended by national guidelines [21]. Nevertheless, increased sensitivity might also result in the detection of transient infections [26], often associated with low viral loads [27], that would have otherwise cleared spontaneously, potentially leading to unnecessary follow‐up procedures and patient anxiety. Balancing sensitivity with clinical relevance is essential.

In HPV‐based screening strategies, various triage approaches have been proposed for women testing positive for HR‐HPV, including cytology, HPV partial genotyping, and other biomarkers, used alone or in combination to guide referral to colposcopy [28]. In France, cytology is used as a triage test in women testing positive for HR‐HPV, with referral to colposcopy depending on cytological findings and/or HPV persistence [21]. Accurate HR‐HPV detection is therefore crucial for appropriate management. In this study, the Allplex™ assay detected more HR‐HPV infections in abnormal cytology samples (ASCUS, AGC, ASCH, LSIL), highlighting its clinical potential. An exception was a discordant HSIL case detected only by ELITe, showing that even a more sensitive assay like Allplex^TM^ can occasionally miss infection in high‐grade lesions. These findings underscore the importance of evaluating HPV assays not only for overall detection rates but also according to cytological categories, as detection discrepancies may reflect variations in viral load, lesion severity, and assay‐specific characteristics. Taken together, these findings indicate that differences in analytical sensitivity between assays may influence downstream clinical decisions, including the proportion of women who undergo repeat testing at 1 year or referral to colposcopy. Clarifying these differences may help determine whether implementing the ELITe assay in place of Allplex™ would modify patient management within HPV‐based screening pathways.

Pre‐analytical differences may also have influenced the results. The Allplex™ assay uses a larger input volume for DNA extraction (800 µL) than the ELITe assay (200 µL), representing a fourfold difference in sample input. Assuming comparable extraction efficiency, such a difference would theoretically correspond to a reduction of up to 1 cycle in Ct values. This estimation is consistent with the Bland–Altman analysis of 199 concordant detections (excluding multiple infections involving HR1 and/or HR2 due to grouped detection with the ELITe), which showed a mean Ct difference of −1.93 ± 3.43, with Allplex™ generally reporting lower Ct values. A moderate correlation between Ct values from the two assays was also observed (R² = 0.54, *p* < 0.0001), indicating consistent but non‐equivalent quantitative performance.

To assess whether discordant results could be linked to sample DNA quality or quantity, IC Ct values were compared between concordant and discordant results for each assay. For the ELITe assay, no significant difference was observed, indicating comparable cellularity and DNA quality across sample groups. For the Allplex™ assay, concordant samples had slightly higher IC Ct values than discordant samples, with a statistically significant difference. This difference suggests that discordant samples did not have reduced DNA quality or quantity, supporting the hypothesis that discrepancies are unlikely to result from suboptimal sample quality. This finding may reflect a technical effect related to PCR competition: in samples with high HPV DNA levels, amplification of the viral target could reduce IC amplification efficiency, increasing IC Ct values. This PCR competition has already been observed for other multiplex PCR and could contribute to the observed differences [29]. Overall, these results suggest that discrepancies are more likely attributable to intrinsic assay characteristics than to sample quality.

Methodologically, this study can also be discussed in light of the international framework for HPV assay validation proposed by Meijer *et al.* [17]. According to these criteria, HPV assays intended for primary cervical screening must demonstrate a relative clinical sensitivity for CIN2+ detection of at least 90% and a relative specificity of at least 98% compared with a clinically validated reference assay, such as Hybrid Capture 2, using samples from a population‐based screening cohort. Intra‐ and inter‐laboratory reproducibility should also reach at least 87% agreement (*κ* ≥ 0.5) on a series of 500 samples, including 30% positives. The present study was not intended as a full clinical validation, since it did not include histopathological endpoints (CIN2 + ) or inter‐laboratory testing. Nevertheless, it fulfills several analytical principles of this framework. The comparative approach using two assays targeting the same HR‐HPV genotypes is consistent with the requirement of performance evaluation against a clinically validated comparator assay. In addition, the study relied on routine cervical samples representative of women undergoing screening in a real‐life setting, thus reflecting the population targeted by Meijer's criteria. Finally, both positive and negative samples were included, allowing the assessment of analytical agreement, relative detection rates, and genotype‐specific performance across clinically relevant cytological categories.

Although not addressing all validation criteria, including clinical sensitivity and specificity for CIN2+ detection as well as intra‐ and inter‐laboratory reproducibility, the study design ensured methodological robustness by minimizing potential biases. In particular, potential pre‐analytical bias was controlled, as storage conditions were taken into account in the study design. Samples collected in 2023 were stored at −20°C and tested with both assays within the same period, limiting storage‐related bias, whereas samples collected from 2024 onward were stored at +4°C before ELITe testing. All discordant results were subsequently retested with the Allplex™ assay after ELITe analysis on samples preserved under the same conditions, ensuring that observed discrepancies were not related to storage.

Furthermore, this study included a large sample size (*n* = 491 valid samples) and at least seven positive samples per genotype, ensuring broad representation across HPV targets. Allplex™ HPV28 (set A) results were interpreted using the Ct cut‐offs defined for the Allplex™ HPV HR assay, supporting their applicability for cervical cancer screening. These elements strengthen the analytical reliability and clinical relevance of the comparison.

However, this study has some limitations. First, the ELITe assay reports certain genotypes in pooled groups, restricting genotype‐specific comparisons, such as the proportion of single versus multiple infections or the association between discordances and infection status. Second, no clinical follow‐up or histopathological endpoints were available to assess the prognostic value of discordant results. While Ct values may provide useful insight, the clinical significance of low viral load detection remains uncertain. Third, 27% of samples had abnormal cytology, a higher proportion than in unselected screening populations, which may have increased agreement between assays. As suggested by Rebolj *et al.*, women with cytological abnormalities often have higher viral loads, whereas unselected screening samples are more heterogeneous, allowing low‐viral‐load cases to influence disagreement rates [30]. Fourth, no cervical cancer samples were included, limiting the comparative evaluation for high‐grade lesions and invasive disease. Fifth, the analysis of discordant results based on Ct was performed using the values from each respective assay (Allplex™ for ELITe − /Allplex+ and ELITe for ELITe + /Allplex−), even though these values are not directly comparable. A third method could have provided an additional result to help resolve discordant cases and allowed Ct values to be compared using the same technique. Sixth, all invalid ELITe results were obtained from samples stored at +4°C before testing. Although these samples were not retested, this observation may suggest a potential impact of prolonged storage at +4°C on DNA integrity, possibly leading to amplification failure with the ELITe assay. Finally, while ASCUS cases were well represented, the low numbers of samples in other abnormal cytology categories (AGC/ASCH/LSIL/HSIL) may limit the robustness of stratified analyses.

In conclusion, the Allplex™ HPV HR assay demonstrated higher analytical sensitivity than the ELITe assay, particularly for HR1 genotypes (notably HPV52), as well as HPV18 and HPV16, and more frequently identified additional HR‐HPV‐positive cases, including in cytologically normal or borderline samples. This enhanced detection includes HPV16 and HPV18, the genotypes most frequently associated with cervical cancer, thereby reinforcing the clinical relevance of the Allplex™ assay in a screening context. These findings underscore the potential impact of assay design and pre‐analytical variables—such as sample input volume and DNA extraction protocols—on HPV detection performance. While the clinical implications of detecting low viral load infections remain to be established, these results highlight the importance of considering assay performance characteristics when interpreting HPV test results. Future studies combining virological, cytological, and histopathological data will be essential to define the optimal balance between analytical sensitivity and clinical utility.

## Author Contributions

Conceptualization and study design: Sarah Mafi and Sébastien Hantz, Experimental work: Julie Delpont and Amandine Bigot, Data analysis and preparation of figures: Sarah Mafi, Julie Delpont, and Sébastien Hantz, Manuscript drafting: Sarah Mafi, Critical revision of the manuscript: Sébastien Hantz. All authors read and approved the final version of the manuscript.

## Funding

The authors have nothing to report.

## Ethics Statement

This study was conducted in accordance with the institution's procedure (USA‐P‐015 B) for the reuse of health data for research purposes in France. Patients were informed of the possibility of reuse of their health data and biological samples for research purposes and could object to this. All data were fully anonymized, with no information that could identify individual participants during or after data collection. Data were accessed for research purposes from January 1, 2023, to March 31, 2025.

## Conflicts of Interest

The authors declare no conflicts of interest.

## Permission to Reproduce Material

No copyrighted material from other sources was reproduced.

## Supporting information

Supporting File

## Data Availability

The data that support the findings of this study are available from the corresponding author upon reasonable request. All relevant data are within the manuscript and its Supporting Information files.
