# Supplementary material for: Comparative Evaluation of HPV PLUS ELITe MGB® and Allplex™ HPV HR Assays for High‐Risk Human Papillomavirus Genotyping in Cervical Samples
Source: J Med Virol. 2026 May 16;98:e70954. doi: 10.1002/jmv.70954 (PMC13179780; doi:10.1002/jmv.70954)
Supplement: Supplementary file 1 — Supporting File [file JMV-98-e70954-s001.docx]

**Supplementary Table 1. HR-HPV genotype distribution according to infection status (single vs. multiple) based on initial Allplex™ HPV28 results interpreted with Allplex™ HPV HR assay cut-offs.**

| **Genotype** | **Single infection (n)** | **Multiple infection (n)** | **Total (n)** |
| --- | --- | --- | --- |
| HPV 16 | 41 | 10 | 51* |
| HPV 18 | 31 | 6 | 37 |
| HPV 31 | 26 | 9 | 35 |
| HPV 45 | 18 | 9 | 27 |
| HPV33 (HR1) | 19 | 2 | 21 |
| HPV52 (HR1) | 20 | 6 | 26 |
| HPV58 (HR1) | 18 | 2 | 20 |
| HPV35 (HR2) | 3 | 4 | 7 |
| HPV39 (HR2) | 8 | 1 | 9 |
| HPV51 (HR2) | 4 | 4 | 8 |
| HPV56 (HR2) | 4 | 3 | 7 |
| HPV59 (HR2) | 5 | 3 | 8 |
| HPV66 (HR2) | 5 | 6 | 11 |
| HPV68 (HR2) | 3 | 5 | 8 |

**A total of 275 HR-HPV genotypes were detected across 236 HR-HPV–positive samples.**

**One HPV16 case was excluded due to invalid ELITe result (n=50 in comparative analysis).*
